# The influence of the *TNFα* rs1800629 polymorphism on some inflammatory biomarkers in 45-60-year-old women with metabolic syndrome

**DOI:** 10.18632/aging.101600

**Published:** 2018-10-31

**Authors:** Małgorzata Szkup, Elżbieta Chełmecka, Anna Lubkowska, Aleksander Jerzy Owczarek, Elżbieta Grochans

**Affiliations:** 1 Department of Nursing, Pomeranian Medical University in Szczecin, 71-210 Szczecin, Poland; 2 Department of Statistics, Department of Instrumental Analysis, School of Pharmacy with the Division of Laboratory Medicine in Sosnowiec, Medical University of Silesia, Katowice, 41-200 Sosnowiec, Poland; 3 Department of Functional Diagnostics and Physical Medicine, Pomeranian Medical University in Szczecin, 71-210 Szczecin, Poland

**Keywords:** metabolic syndrome, *TNFα* polymorphism, proinflammatory cytokines, interleukins, women's health

## Abstract

Introduction: There are reports that the *TNFα* gene (rs1800629) can be involved in the pathogenesis of metabolic syndrome through an increased production of pro-inflammatory cytokines. Therefore, we have decided to search for the relationship between the *TNFα* gene polymorphisms and serum levels of proinflammatory cytokines (IL-1α, IL-1β, IL-6, TNFα, IFNγ) and CRP in women with metabolic syndrome.

Material and methods: The study sample consisted of 416 women aged 45-60 years, including 118 with metabolic syndrome. The participants were surveyed and subjected to anthropometric, biochemical and genetic analysis.

Results: We noticed that in the group meeting the criteria for metabolic syndrome, the G/G genotype of the *TNFα* gene was related to higher IL-6 levels than in the remainder group. The carriers of the A/G genotype in the metabolic syndrome group had significantly higher levels of IFNγ than those in the group without this syndrome. CRP was significantly higher in the group with metabolic syndrome, irrespective of the women’s genotypes.

Conclusions: The upregulation of IFNγ and IL-6 and CRP suggests that autoinflammatory process may play a significant role in the pathogenesis of metabolic syndrome. However, a direct relationship between the *TNFα* gene polymorphisms and inflammatory biomarkers analyzed in our study was not confirmed.

## Introduction

The problem of Metabolic syndrome (MetS) affects 20-30% of the middle-aged population, increasing a risk of cardiovascular diseases and premature death. The criteria for MetS diagnosis include three out of five markers: abdominal obesity (manifested by an increased waist size), impaired carbohydrate metabolism (fasting hyperglycemia), high blood pressure and dyslipidemia (elevated levels of triglycerides (TG) and decreased levels of high-density lipoprotein (HDL)) [1–3].

Some studies suggest that most symptoms of MetS are associated with an increased production of C-reactive protein (CRP) and pro-inflammatory cytokines (PICs) ― especially interferon gamma (IFNγ) and tumor necrosis factor*-*alpha (TNFα) ― as well as a higher incidence of highly productive alleles of genes contributing to PICs production [4]. The pathogenesis of MetS is currently believed to be underlain not only by environmental but also genetic factors [5] and chronic subclinical inflammation [6]. However, the exact inflammatory mechanism in the disorder manifestation has not yet been established. Genetic factors contributing to the development of MetS include the polymorphic variants of the genes whose expression affects particular components of MetS, such as obesity, insulin resistance, carbohydrate and lipid metabolism disorders [7].

The *TNFα* (-308 A/G) rs1800629 polymorphic gene has an influence on the production of TNFα. It has been noticed that Healthy carriers of the A allele have higher levels of TNFα than their counterparts with the low-production G allele [8]. The presence of the A allele of the *TNFα* gene, which is a high-production allele, increases the binding of a transcription factor to the promoter region of the *TNFα* gene, thereby altering its expression [9]. More than that, it positively correlates with the occurrence of obesity, high systolic blood pressure, and the plasma insulin level. These results support the hypothesis that the *TNFα* gene can be involved in the pathogenesis of metabolic syndrome [10].

TNFα is a proinflammatory cytokine with pleiotropic biological effects, which in inflammation, together with its receptors (TNFR1 and TNFR2) activates caspase cascade and transcription factors (AP1 and NF-κβ). This, in turn, induces cytokines that trigger immune response, which includes the activation of TNFα, Interleukin 1 (IL-1) and Interleukin 6 (IL-6) [9].

Circulating cytokines interact with specific receptors on various cell types, and activate the JAK-STAT, NF-κB, and SMAD signaling pathways, leading to an inflammatory response involving cell adhesion, acute phase proteins, permeability and apoptosis [11]. Mediators, such as: Interleukin 1α (IL-1α), Interleukin 1β (IL-1β), IL-6, TNFα, IFNγ may produce a proartherogenic effect. It is believed that taken together, hypercholesterolemia and an inflammatory factor form a basis for the development of atherosclerosis [12]. Furthermore, recent studies show that IFNγ may considerably contribute to inflammatory response associated with obesity [13]. There is also a relationship between high TNFα levels and the presence of MetS components in elderly people without a diagnosis of type 2 diabetes or cardiovascular disease [14]. PICs cause exacerbation of insulin resistance and MetS-related symptoms [15]. Presumably, TNFα reduces the activity of insulin, which may lead to the development of type 2 diabetes [16].

A special liver-derived pattern-recognition molecule, CRP belongs to inflammatory factors that contribute to host defense [17]. An increase in CRP levels can lead to greater secretion of pro-inflammatory factors. As an effect, it can cause serine phosphorylation of insulin receptor substrate proteins, yielding in a decrease in insulin signaling, and ultimately increasing insulin resistance [18]. Elevated CRP levels also enhance the risk of cardiovascular disease by inducing the adhesion molecule expression in endothelial cells [19].

In this study, we decided to assess the genetic role of the *TNFα* gene rs1800629, and its effect on the expression of important components of the inflammatory pathway. We assumed that the *TNFα* gene polymorphism may affect the production and expression of proinflammatory cytokines (IL-1α, IL-1β, IL-6, TNFα, IFNγ) and CRP in patients with MetS. The risk of MetS has been noticed to significantly increase in the peri- and postmenopausal periods, irrespective of age and other commonly known risk factors for cardiovascular disease [20,21]. It is suggested that a decline in ovarian function with menopause is associated with a spontaneous increase in the levels of proinflammatory cytokines. In the light of these data, we conducted our study among 45-60-year-old women with especially high risk of MetS.

The purpose of this study was to seek the relationship between the *TNFα* gene polymorphisms and serum levels of proinflammatory cytokines (IL-1α, IL-1β, IL-6, TNFα, IFNγ) and CRP in 45-60-year-old women with MetS.

## RESULTS

The mean age of the participants with SD was 53 ± 5 years. 33.6% of the respondents had primary education, 27.7% had higher education, 26.9% had secondary education, and 11.8% had vocational education. The most numerous of the women (44.8%) were those living in big cities (more than 100,000 residents), 39.4% lived in rural areas, and the rest in smaller cities. The majority of the women (82.7%) were married, 6.2% cohabited with their partners, and 11.6% were single. The MetS+ group comprised of 118 women (28.37% of all participants). Comparative analysis of the MetS+ and MetS- groups revealed statistically significant differences in all five MetS components according to the diagnostic criteria of the International Diabetes Federation (IDF) from 2009 [3]. All MetS components were significantly more common in MetS+ group, however, what was interesting, even in the MetS- group the mean waist size was bigger than normal (≥ 80 cm). Analysis of the mean values for MetS components showed that in the MetS+ group the norms recommended by the IDF were exceeded for waist size, fasting glycemia, and systolic blood pressure. The MetS markers that were most often observed in the MetS+ group were big waist size (82.22% of all women in the group) and hypertension (82.2%). The women with MetS were also older compared with those without MetS.

Analysis of biochemical parameters demonstrated higher CRP and IL-6 levels in the group with MetS. There were no statistically significant differences in the levels of other PICs (Table 1).

**Table 1:** Characteristics of the study sample with regard to a division into MetS+ and MetS- groups.

	***MetS+*** *N = 118*	***MetS–*** *N = 298*	*p*
**MetS components**			
waist size [cm] MetS symptom def. ― waist size [cm]; N (%) fasting glycemia [mg/dl] MetS symptom def. ― hyperglycemia; N (%) TG [mg/dl] MetS symptom definition ― TG; N (%) HDL [mg/dl] MetS symptom definition ― HDL; N (%) systolic blood pressure [mmHg] diastolic blood pressure [mmHg] MetS symptom def. ― hypertension; N (%)	93.3 ± 11.0 97 (82.2) 100.8 (86.9 – 119.0) 60 (50.8) 137.6 (102.0 – 189.8) 75 (63.6) 56.5 ± 16.8 61 (51.7) 137.2 ± 15.3 83.9 ± 9.4 97 (82.2)	85.4 ± 11.2 62 (20.8) 83.2 (77.4 – 90.7) 14 (4.7) 84.8 (65.0 – 112.1) 32 (10.7) 70.0 ± 16.0 21 (7.1) 119.1 ± 14.8 75.9 ± 9.7 62 (20.8)	**< 0.001** **< 0.001** **< 0.001** **< 0.001** **< 0.001** **< 0.001** **< 0.001** **< 0.001** **< 0.001** **< 0.001** **< 0.001**
**PICs and CRP**			
IL-1α [pg/ml]	1.85 (1.46 – 2.42)	2.09 (1.50 – 2.59)	0.079
IL-1β [pg/ml]	13.64 (5.60 – 102.00)	12.39 (3.41 – 218.30)	0.801
IL-6 [pg/ml]	11.23 (5.51 – 34.47)	8.21(3.65 – 21.46)	**< 0.05**
TNFα [pg/ml]	4.06 (2.00 – 6.85)	3.70 (1.94 –6.39)	0.495
IFNγ [IU/ml]	0.04 (0.03 – 0.21)	0.05 (0.03 – 0.14)	0.936
CRP [mg/l]	3.2 (1.80 – 5.50)	1.90 (1.30 –3.20)	**< 0.001**

mean ± standard deviation; median (lower quartile – upper quartile); p ― significance level. PICs – proinflammatory cytokines; CRP – C-reactive protein; IL – interleukin; TNFα – tumor necrosis factor α; IFNγ – interferon γ.

In the whole study sample, the G/G genotype was the most frequent of the *TNFα* gene variants (75%). The G allele was observed in the vast majority of the women: 85% of the MetS+ group and 86% of the MetS- group. There were no statistically significant differences in the distribution of the genotypes and alleles between both groups (Table 2).

**Table 2:** Analysis of the distribution of the *TNFα* gene rs1800629 polymorphisms with regard to MetS.

	***TNF****α ****genotype***			***TNF****α ****allele***	
	**A/A** **n (%)**	**G/A** **n (%)**	**G/G** **n (%)**	**A allele** **n (%)**	**G allele** **n (%)**
**MetS+**	2 (2)	31 (26)	85 (72)	35 (15)	201 (85)
**MetS–**	9 (3)	63 (21)	226 (76)	81 (14)	515 (86)
**Σ**	11 (3)	94 (22)	311 (75)		
**p**	p = 0.425			p = 0.641	

n ― number of cases; **Σ** ― sum of cases; p ― significance level.

As the last stage of the study, we tested the association between the *TNFα* gene rs1800629 polymorphisms and serum PIC levels in relation to MetS. We noticed that in the MetS+ group, the G/G genotype of the *TNFα* gene was accompanied by higher IL-6 levels than in the MetS- group. At the same time, the carriers of the A/G genotype in the MetS+ group had significantly higher IFNγ levels than those in the MetS- group. CRP was visibly higher in the group with MetS, irrespective of the women’s genotypes.

The levels of IL-1α, IL-1β, and TNFα were not statistically significantly related to any of the tested genotypes of the *TNFα* gene in any of the MetS groups (Table 3).

**Table 3:** Analysis of the relationships between the *TNFα* gene rs1800629 polymorphisms and the levels of IL-1α, IL-1β, IL-6, TNFα, IFNγ in relation to MetS.

***TNFα*** ***genotype***	***MetS+***	***MetS-***		
	Mean value (SE); N	Mean value (SE); N	Δ	± 95% CI
log_10_ (IL-1α [pg/ml])				
G/G	0.324 (0.042); 85	0.385 (0.028); 226	-0.062	-0.162 ÷ 0.038
A/G	0.292 (0.044); 31	0.368 (0.054); 63	-0.076	-0.248 ÷ 0.097
A/A	0.286 (0.019); 2	0.279 (0.041); 9	0.006	-0.608 ÷ 0.620
log_10_ (IL-1β [pg/ml])				
G/G	1.313 (0.109); 74	1.338 (0.078); 187	-0.0251	-0.303 ÷ 0.253
A/G	1.332 (0.163); 24	1.256 (0.153); 49	0.076	-0.428 ÷ 0.581
A/A	2.030 (0.937); 2	1.206 (0.467); 7	0.824	-0.798 ÷ 2.446
log_10_ (IL-6 [pg/ml])				
G/G*	**1.256 (0.088); 75**	**0.996 (0.040); 198**	**0.260**	**0.089 ÷ 0.431**
A/G	1.123 (0.107); 27	1.091 (0.099); 59	0.032	-0.261 ÷ 0.325
A/A	1.735 (0.666); 2	0.883 (0.165); 9	0.852	-0.134 ÷ 1.838
log_10_ (TNFα [pg/ml])				
G/G	0.621 (0.059); 65	0.546 (0.034); 191	0.075	-0.056 ÷ 0.206
A/G	0.531 (0.084); 25	0.509 (0.056); 54	0.022	-0.199 ÷ 0.243
A/A	–	0.604 (0.196); 8	–	–
log_10_ (INFγ [IU/ml])				
G/G	-1.207 (0.090); 36	-1.075 (0.047); 121	-0.132	-0.334 ÷ 0.071
A/G*	**-0.681 (0.221); 16**	**-1.243 (0.073); 38**	**0.562**	**0.244 ÷ 0.879**
A/A	–	-1.187 (0.198); 6	–	–
log_10_(CRP [mg/l])				
G/G*	**0.550 (0.043); 69**	**0.337 (0.022); 128**	**0.214**	**0.126 ÷ 0.301**
A/G*	**0.488 (0.075); 23**	**0.321 (0.044); 39**	**0.167**	**0.014 ÷ 0.321**
A/A	–	0.307 (0.109); 8	–	–

N ― number of cases; SE ― standard error of the mean; Δ – mean difference between groups; ± 95% CI ― 95% confidence interval; CRP – C-reactive protein; IL – interleukin; TNFα – tumor necrosis factor α; IFNγ – interferon γ. * – statistically significant based on the ± 95% CI

## DISCUSSION

The research on mice has confirmed that greater amount of white adipose tissue contributes to an increase in the levels of IL-1, IL-6, TNFα, and IFNγ. The production of IFNγ in obese mice was higher than in the control group, and was accompanied by IFNγ receptor deficiency [22]. Injection of TNFα or IL-6 given to pregnant rats caused an increase in the mass of adipose tissue in their offspring by 30-40% [23]. The production of IFNγ in women over 40 and in the postmenopausal period is considerably higher than in younger patients [24]. In our study, significantly higher IL-6 levels were only found in the women who met the criteria for inclusion in the MetS+ group. This observation corresponds with available results of other authors, who not only reported relationships between the levels of IL-6 and the occurrence of MetS, but also noticed that higher IL-6 levels entailed more severe manifestation of MetS symptoms (hypertriglicerydemia, fasting glycemia, and hypertension) [25–27]. Chedraui et al. demonstrated the relationship between elevated IL-6 levels and abdominal obesity, low HDL levels, and high TG levels in postmenopausal women [26], while Indulekha et al. found the connection between IL-6 levels and insulin resistance [27]. The levels of other PICs analyzed in our study were similar in all participants. In our investigation, CRP was significantly higher in the women with MetS. Similar outcomes were obtained by Also Ren et al. who established that higher CRP levels were linked to an increased prevalence of MetS and four out of its five components [17]. These results have been confirmed by other authors analyzing patients with type 2 diabetes [28] and low-income dwellers of rural areas [29].

The study conducted by Pausov et al. on rats suggests that the region of chromosome 6 in the *TNFα* (-308) gene is involved in the pathogenesis of obesity and obesity-related hypertension. It also has an influence on adiposity, glucose tolerance, serum leptin levels, and blood pressure, but only when the rats are given a high-fat diet [30]. The aim of the study conducted by de Luis et al. was to investigate the influence of the *TNFα* gene polymorphism on insulin resistance and weight loss secondary to a hypocaloric diet in obese patients. The obese patients with the A allele of *TNFα* gene had a higher initial weight before starting and after completing treatment than the carriers of the G allele. Presumably the patients with the low*-*production alleles showed a better metabolic response than those with the high-production A allele [31]. Our study did not demonstrate any differences in the distribution of the genotypes and alleles of the *TNFα* gene between the MetS+ and the MetS- groups.

Conclusions from the meta-analysis performed by Sookoian et al. show that the risk of health problems, such as obesity, high systolic blood pressure, and high plasma insulin level, increases by 23% for carriers of the A allele of the *TNFα* gene. These results support the hypothesis that the *TNFα* gene could be involved in the pathogenesis of MetS [10]. Rangel-Zúñig et al. demonstrated that activation of the proinflammatory status in people with the G/G genotype is greater than in A-allele carriers. It could induce DNA damage, especially in the telomeric sequence, leading to a decrease in the telomere length. According to these authors, this effect may boost the risk of the development of age-related diseases [32]. Out of the MetS patients qualified for the study on the influence of Mediterranean diet on triglyceride metabolism and inflammation status, the G/G subjects showed higher fasting and postprandial TG and CRP plasma concentrations than the carriers of the minor A allele (G/A + A/A) [33]. In our study, the carriers of the G/G genotype of the *TNFα* gene from the MetS+ group had higher IL-6 levels than their counterparts from the MetS- group. What is more, the MetS+ women with the A/G genotype had higher levels of IFNγ than those without MetS.

We found that the distribution of the genotypes and alleles was very similar in both groups, which indicates that there is no direct relationship between the *TNFα* gene rs1800629 polymorphism and the occurrence of MetS. Due to a small number of patients with the A/A genotype, the results obtained for this subgroup could not be analyzed, and thus no conclusions could be drawn as for the influence of this genotype on the levels of PICs and CRP.

IL-6 is often secreted by M1 macrophages and plays a role in the natural inflammatory response [34]. An increase in the number of M1 macrophages within adipose tissue in MetS can result in higher secretion of IL-6 from adipose tissue, and consequently lead to insulin resistance. IL-6 contributes to endothelial cell damage in blood vessels, causing arthrosclerosis. More than that, IL-6 can cause abnormalities in insulin signaling cascade, insulin action and glucose metabolism due to aberrant insulin receptor activation. Our preliminary findings described in this study can be supported by different authors [25–27,35]. CRP, on the other hand, is known as a marker of inflammation that is strongly related to obesity [36]. There are several studies to support our findings that elevated CRP levels are related to MetS [37,38].

Our investigation of the role of inflammatory pathway components in the development of MetS in relation to the *TNFα* gene polymorphisms revealed upregulation of IFNγ, IL-6, and CRP, which may suggest that autoinflammation plays a part in the disorder pathogenesis. However, a direct relationship between the *TNFα* gene polymorphisms and inflammatory biomarkers analyzed in our study was not confirmed. Therefore, clinical investigators are encouraged to assess markers of inflammation (especially IL-6 and CRP) as the risk factors for MetS in perimenopausal women. Close association between these markers and MetS implies that they play an important part in the development of MetS, and so they would be good components of the biomarker panel.

## MATERIALS AND METHODS

The study sample consisted of 416 women, including 118 (28.4%) with MetS, from the general population of the Westpomeranian Province (Poland). The inclusion criteria were: female sex, 45-60 years of age (mean with SD: 53 ± 5 years), the lack of current inflammatory, psychiatric or cancerous diseases, and informed written consent to take part in the study. Recruitment was performed based on information posters in public places and advertisement in local papers. The study sample characteristics are presented in Table 1.

### Ethics Statement

The investigation was conducted in compliance with ethical standards, the Declaration of Helsinki, and national and international guidelines. The protocol of the study was approved by the Bioethical Commission of the Pomeranian Medical University of Szczecin, Poland (permission number KB-0012/181/13). The participants’ informed consent has been obtained.

### Description of the research procedure

The research procedure consists of four stages: interview, anthropometric measurements, genetic analysis, and measurement of PICs levels.

At the first stage of the procedure, we asked about basic sociodemographic data (age, place of residence, professional activity, education, marital status), and information concerning pharmacotherapy for hypertension, hypertriglyceridemia, hyperglycemia, and low HDL levels. The patients were also asked about their current inflammatory, psychiatric and cancerous diseases.

Next, blood pressure was gauged in a sitting position using a manual manometer by registered nurses. The cuff of the manometer was matched to the arm circumference and wrapped snugly around the patient's right upper arm at the heart level. Waist was measured in a standing position between the lower rib margin and the upper margin of the iliac crest at the end of a gentle exhalation.

As the next step, venous blood was collected from each volunteer after overnight fasting, between 7.00 and 9.30 in the morning, after a 10 min rest in a sitting position, from the antecubital vein using Vacutainer tubes (Sarstedt, Germany), separately into two tubes: one with 1 g/L K2 EDTA and the other for biochemical analysis of serum (7 mL).

The blood was collected in accordance with the relevant rules and procedures concerning collecting, storing, and transporting biological material. The levels of fasting glycemia, TG and HDL were determined. Next, DNA was isolated for genetic analysis of the *TNFα* gene rs1800629 polymorphisms. The rest of the blood was used to assess PICs levels.

All participants were divided into two groups: the first group included women who met the criteria for MetS (MetS+ group) according to the IDF diagnostic criteria from 2009 [21], and the second group comprised of women without MetS (MetS- group). The women were qualified to MetS+ group if they had at least three out of five symptoms: waist size ≥ 80 cm; fasting glycemia ≥ 100 mg/dl (5.6 mmol/l) or related pharmacotherapy; TG level ≥ 150 mg/dl (1.7 mmol/l) or related pharmacotherapy; HDL cholesterol level ≤ 50 mg/dl (1.3 mmol/l) or related pharmacotherapy; blood pressure: systolic blood pressure ≥ 130 and/or diastolic blood pressure ≥ 85 mmHg or related pharmacotherapy.

### DNA isolation and the TNFα gene polymorphism genotyping

Genomic DNA was isolated from the whole blood according to standard salting procedures [39].

A polymorphism in the *TNFα* gene (rs1800629 A/G) was genotyped with the fluorescence resonance energy transfer method Real-Time PCR using the Light Cycler II. The following conditions were applied: polymerase chain reaction (PCR) was performed with 50 ng DNA in a total volume of 20 ml containing 2 ml reaction mix, 0.5 mM of each primer, 0.2 mM of each hybridization probe and 2 mM MgCl2 according to the manufacturer’s instructions for 35 cycles of denaturation (95°C for 10min), annealing (60°C for 10 sec) and extension (72°C for 15 sec). After amplification, a melting curve was generated by holding the reaction at 40°C for 20 seconds and then heating slowly to 85°C. The LightSNiP primers and probe for rs1800629 were used in the assay (TIB MOLBIOL GmbH, Germany). The fluorescence signal was plotted against temperature to give melting curves for each sample.

### The measurement of PICs levels

The serum levels of IL-1α, IL-1β, IL-6, TNFα and IFNγ were measured by immune-enzymatic assays using commercially available enzyme-linked immunosorbent (ELISA) kits according to the manufacturer's protocol. The serum levels of IL-1α, IL-1β, IL-6, TNFα and IFNγ were measured by immune-enzymatic assays using commercially available ELISA kits (DRG, Germany). The IL-1α assay sensitivity was 1.1 pg/ml, intra- and inter-assay CVs were < 5.4% and < 10%, respectively. The IL-6 assay sensitivity was 2 pg/ml, intra- and inter-assay CVs were 4.2% and 4.4%, respectively. The IL-1β assay sensitivity was 0.35 pg/ml, intra- and inter-assay CVs were 2.3% and 4.9%, respectively. The IFNγ assay sensitivity was 0.03 IU/mL, intra- and inter-assay CVs were 3.2% and 5.8%, respectively. The TNFα assay sensitivity was 0.7 pg/ml, intra- and inter-assay CVs were 6.3% and 4.5%, respectively.

### Statistical analysis

Statistical analysis was performed using Statistica 13 PL (TIBCO, Palo Alto, USA) and R (CRAN) software. Statistical significance was set at p < 0.05. All tests were two-tailed. No data imputation has been done. Interval data were expressed as a mean ± standard deviation in the case of normal distribution, and as a median / lower–upper quartile in the case of data with skewed or non-normal distribution. The distribution of variables was evaluated by the Shapiro-Wilk test and the quantile-quantile plot. The homogeneity of variance was assessed by the Fisher-Snedecor test. In the case of skewed data distribution, logarithmic transformation was done before analysis. The following tests were used to verify hypotheses: the parametric test for two independent samples (Student’s t-test) in the case of normal distribution or after logarithmic transformation, and the Mann-Whitney U test if the distribution was not normal. Nominal and ordinal data were compared with the χ^2^ test. The association analysis between the *TNFα* gene rs1800629 polymorphisms an proinflammatory cytokines were done on the basis of generalized linear models (for quantitative traits – QTL analysis) with *SNPassoc* package.
